# Photothermal heating and heat transfer analysis of anodic aluminum oxide with high optical absorptance

**DOI:** 10.1515/nanoph-2022-0244

**Published:** 2022-06-14

**Authors:** Nicholaus Kevin Tanjaya, Manpreet Kaur, Tadaaki Nagao, Satoshi Ishii

**Affiliations:** International Center for Materials Nanoarchitectonics (MANA), National Institute for Materials Science (NIMS), Tsukuba, Ibaraki, 305-0044, Japan; Faculty of Pure and Applied Physics, University of Tsukuba, Tsukuba, Ibaraki, 305-8577, Japan; Department of Condensed Matter Physics, Graduate School of Science, Hokkaido University, Sapporo, Hokkaido, 060-0810, Japan; PRESTO, Japan Science and Technology Agency, Kawaguchi, Saitama, 332-0012, Japan

**Keywords:** heat transfer, plasmonic heating, porous structure, titanium nitride

## Abstract

Photothermal heating with metallic nanostructures has the unique property of generating heat at the nanoscale owing to plasmon resonances. In this study, the heat transfer of anodic aluminum oxides (AAOs) coated with plasmonic titanium nitride (TiN) of 80 nm thickness are experimentally, numerically, and analytically studied, wherein TiN photothermally generated heat. High optical absorptance and photothermal heating efficiency are observed for the samples with pore sizes in the range of 161–239 nm, and the sample with the pore size of 239 nm exhibits the highest absorptance and photothermal heating efficiency. In addition, the numerical and analytical heat transfer analyses using the effective thermal conductivities for AAO-TiN samples are in reasonable agreement with experimental results, indicating the validity of effective thermal conductivities, which consider the periodic nature. These results can be extended to design other optically absorbing periodic structures for photothermal heating applications.

## 1 Introduction

Photothermal heating is a phenomenon wherein light energy is converted into thermal energy via absorption. A significant number of studies on photothermal results have been conducted in various disciplines, such as photothermal therapy [1, 2], thermal imaging [3], water evaporator [4–9], nanofabrication [10, 11], microfluid [12–14], and ultrafast molecular diagnosis [15]. In the studies on photothermal heating, the primary focus has been on enhancing the optical absorption to generate heat, either with highly absorbing materials or with high optical absorptance nanostructures.

Among highly absorbing nanomaterials, which include carbon nanoparticles [16, 17] for instance, nanomaterials or nanoparticles with plasmonic properties [2, 4, 5, 8, 18], [19], [20] have attracted significant attention. Localized surface plasmon resonances enhance the optical absorption to generate heat, even at the sub-diffraction-limit. Gold has been primarily used to date among plasmonic materials, owing to its good chemical stability and high photothermal conversion efficiency [1, 3, 21]; however, gold nanostructures typically have narrow absorption spectra.

Recent studies [4, 8, 9, 18, 19, 22] have reported that titanium nitride (TiN) is another plasmonic material with photothermal properties superior to those of gold. Typically, TiN nanoparticles exhibit broader and higher absorption spectra than those of gold nanoparticles, making them better broadband solar absorbers [8, 20, 23]. Another advantage of using TiN in photothermal research is that it exhibits a temperature-dependent Raman shift, which can be used to monitor the temperature changes under optical irradiation [18, 19, 24].

In addition to optical absorptance, the thermal conductivities of the entire sample, including intermediate layers and substrates, also affect the surface temperature increase. One may consider replacing the substrate with a material with lower thermal conductivity (e.g., from silicon (142 W/m/K [25]) to glass (1 W/m/K [26])) or using a membrane as a substrate; however, a dilemma caused by restrictions related to sample fabrication and applications will always be present.

To circumvent this issue, one may consider simultaneously designing a nanostructure with high optical absorptance and low thermal conductivity. A highly absorbing nanostructure generates heat by photothermal effect, where the heat dissipation is suppressed with the effectively low thermal conductivity. A candidate for such nanostructure is porous structures. Compared with highly dense materials, porous materials can simultaneously reduce reflection to increase absorption and heat dissipation by introducing air gaps. While some porous materials have random arrangements of pores [9], others have aligned pores. Anodic aluminum oxide (AAO) is one of the most well-known structures among periodically aligned porous materials, owing to its easy and controllable fabrication. Its periodic nature has the advantage of systematically controlling the effective thermal conductivity [27]. The periodically aligned nature of AAO makes it particularly suitable for solar water desalination [5, 8, 9] by effectively evaporating water through straight capillary channels. Although AAO has been used in photothermal studies, the studies on the quantitative analyses of temperature increase and heat conduction are scarce.

In this study, we performed quantitative heat transfer analyses of photothermally heated AAO coated with TiN with a thickness of 80 nm (TiN-AAO); we performed these analyses experimentally, numerically, and analytically. The top TiN layer acted as an efficient light absorber to enhance the photothermal heating effect. AAOs with various pore sizes and thicknesses were fabricated using an electrochemical method to examine the impact of the geometrical parameters on photothermal heating. A 785 nm focused laser beam was used to locally heat the sample surface, and the temperature increase was characterized using Raman spectroscopy. The analytical method involved solving the heat transfer equation in an anisotropic structure, while the numerical method is based on the finite element method. Both analytical and numerical results reproduced the experimentally observed photothermal heating, suggesting a strong influence of the pore size (not the thickness) on increasing the surface temperatures of relatively thick samples.

## 2 Results and discussion

**Figure 1:**
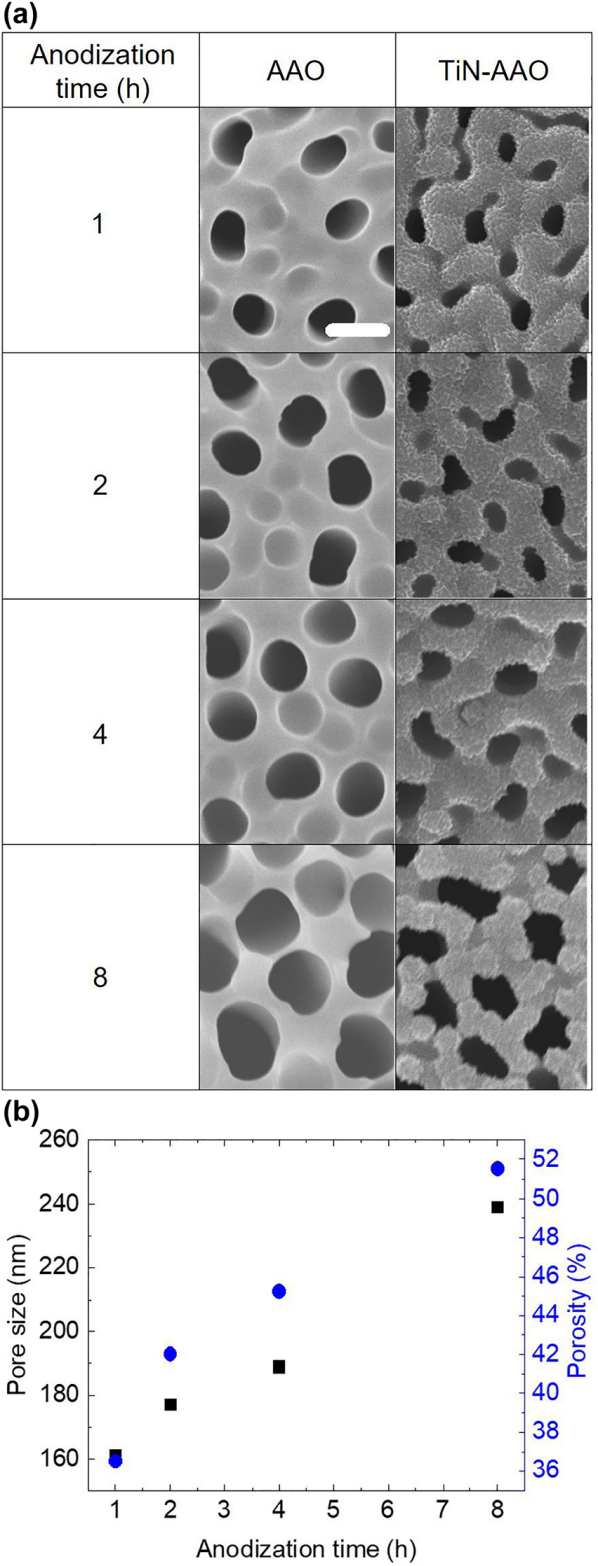
Morphologies of AAO and TiN-AAO after pore-widening for 1 h. (a) SEM images at different anodization times. The scale bar corresponds to 200 nm. (b) Anodization time-dependent pore size (square) and porosity (circle) with a standard deviation error of 20 nm.

AAO samples were fabricated following a previously reported procedure [8, 28] and are detailed in the experimental section. The pore size of AAO was systematically controlled by changing the anodization time from 1 to 8 h and the pore-widening time from 1 to 2 h. It is noteworthy that increasing the pore-widening time above 2 h resulted in interconnected pores, owing to sidewall thinning. Figures 1(a) and S1 present the SEM images of the AAO pores widened for 1 and 2 h, respectively. The SEM images show that the pore size distribution is arranged in a hexagonal lattice as a whole, and TiN-AAO samples exhibit a reduction in the pore size due to TiN filling into the pores. The sample morphologies for the samples pore-widened for 1 h are shown in Figure 1(a). In Figure 1(b), the pore size, which defined by averaging the major and minor axes, indicates a linear increase for a longer anodization time in the range of approximately 161–239 nm. Furthermore, porosity was calculated as the average pore area divided by the average unit cell area (assuming a honeycomb array), and it varied from 36% to 52%. For the AAO samples which were pore-widened for 2 h, the ranges of pore size and porosity are 214–252 nm and 48–52%, respectively.

**Figure 2:**
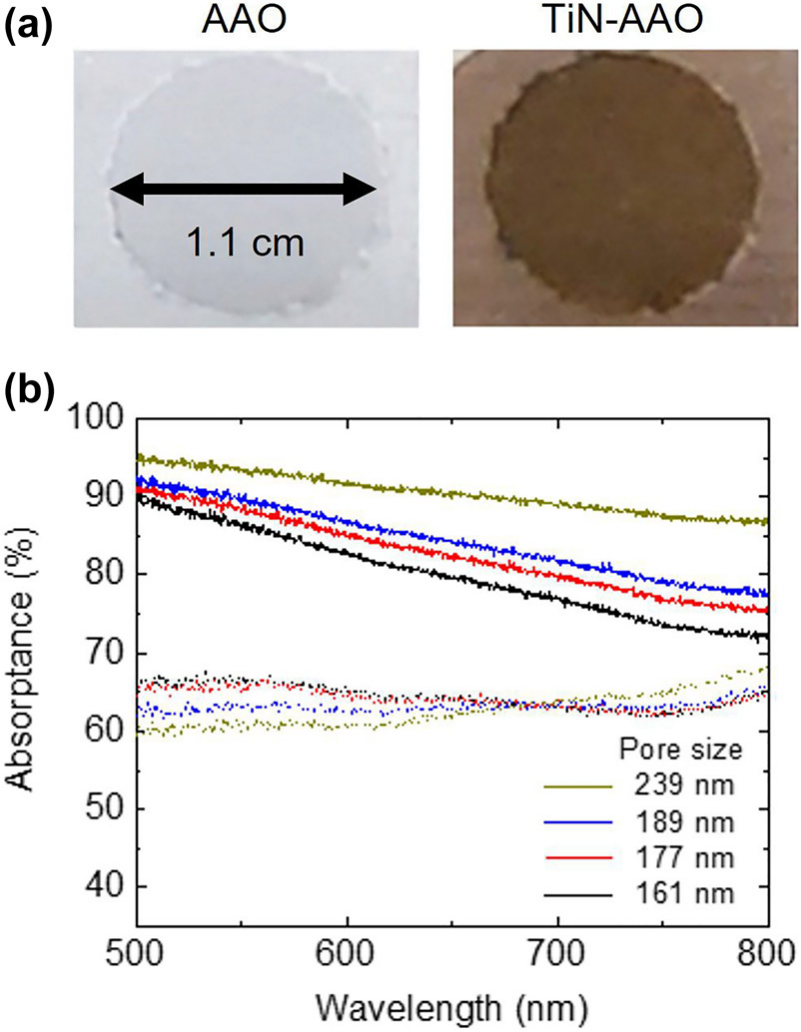
Optical images and absorptance of AAO and TiN-AAO samples. (a) Photographs of the AAO and TiN-AAO samples. Both samples were anodized for 1 h, and pore-widening was conducted for 1 h. The circular areas of AAO are 1 cm^2^ in both samples. (b) Absorptance spectra in the visible range. The bold and dotted lines correspond to the TiN-AAO and AAO samples, respectively.

Using UV–Vis spectroscopy, optical absorptance was calculated by subtracting the diffused reflectance from 100%, where transmittance was 0% because of the thick aluminum (Al) plate at the bottom of AAO, which initially had a thickness of 1 mm. A higher optical absorptance corresponds to a better light-to-heat conversion. The absorptance of AAO and TiN-AAO where the AAOs were pore-widen for 1 h and 2 h are shown in Figures 2 and S2, respectively. As a reference, the absorptance of a planar TiN thin film is shown in Figure S3. As shown in Figure 2, with TiN on AAO, higher absorptance can be observed up to 91% on average for an anodization time of 8 h, indicating that the addition of TiN significantly enhances absorptance. TiN can improve absorptance owing to its plasmonic properties, acting as a light absorber in a broad spectrum. In addition, larger pores exhibited higher absorptance values. It is noteworthy that the AAO without TiN exhibits relatively high absorptance (above 60%). The numerical electromagnetic simulations presented in the Supporting Information (Note 1 and Figure S4) quantitatively reveal that the roughness between the AAO and Al plate was the origin for high absorptance.

**Figure 3:**
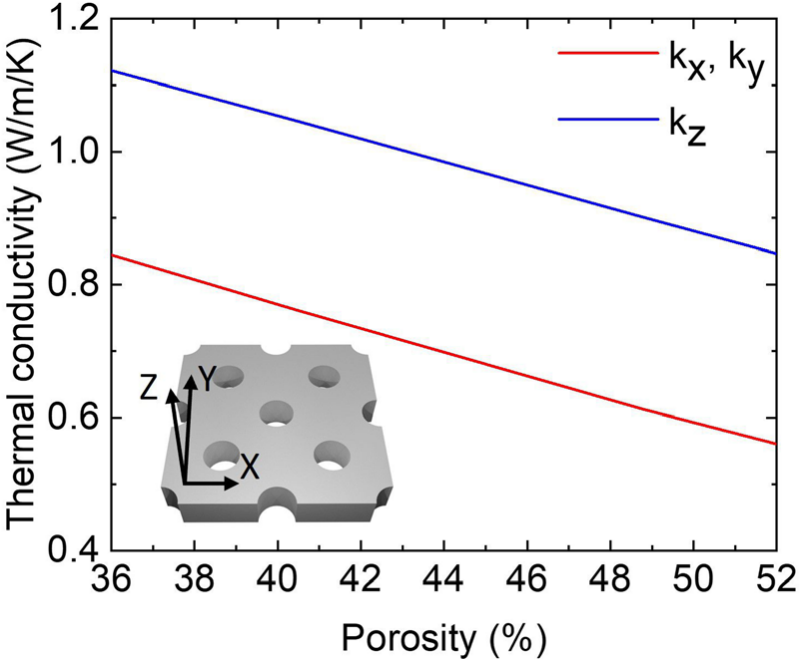
Effective thermal conductivity of AAO with different porosities simulated using the finite element method. Inset: AAO hexagonal lattice schematic.

Effective thermal conductivities were introduced for AAOs comprising alumina and air, and presented in Figure 3. The TiN layer on the AAO has little contribution in the heat conduction, thus not modeled in the effective thermal conductivity. The use of effective thermal conductivities reduced the computational cost of calculating the laser-power-dependent temperature in the numerical heat transfer simulations which are discussed later. From Figure 3, the effective thermal conductivities were in the ranges of 0.85–1.10 and 0.55–0.85 W/m/K for the parallel (*k*_x_ and *k*_y_) and perpendicular (*k*_z_) components, respectively, which are anisotropic. The origin of anisotropy is the vertically aligned pores, which dominate heat conduction in AAO. Another thing that can be inferred from Figure 3 is that a lower effective thermal conductivity can be achieved with a highly porous material. Additionally, simulation results were compared with Maxwell’s effective media theory (Supporting Information, Note 2). The comparison in Figure S5 indicates that AAO thermal conductivity can also be approached using Maxwell’s theory.

**Figure 4:**
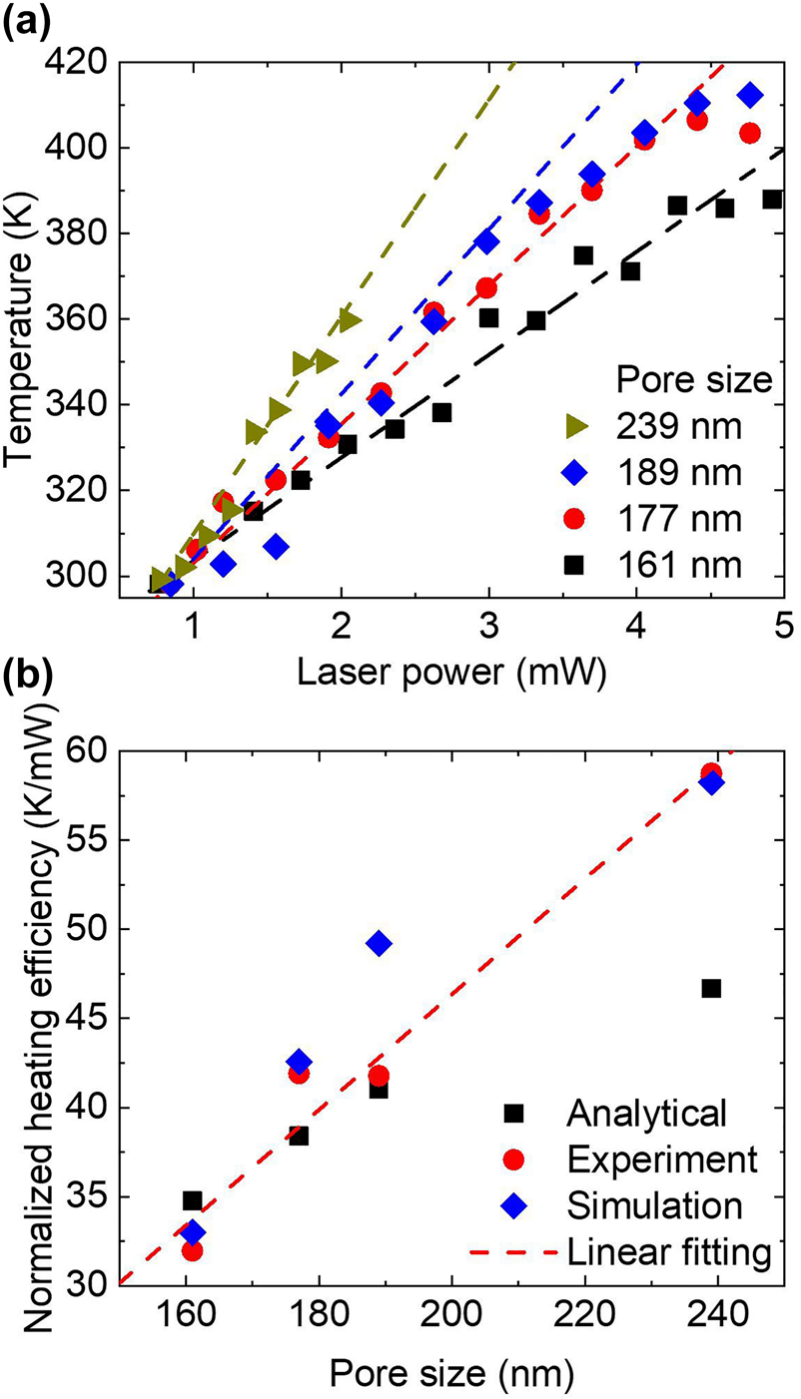
Temperature measurements and heat transfer analyses of photothermally heated TiN-AAO samples. (a) Laser-power-dependent temperature increase from experiments (symbol) and simulations (dashed line). (b) Photothermal heating efficiency of the experiment, simulation, and analytical data where heating efficiency is the slope of power-dependent temperature normalized by absorptance. In subpanels (a) and (b), the TiN-AAO samples were pore-widen for 1 h. The linear fitting in subpanels (b) were fitted from the experiment results.

Laser-power-dependent temperature increase was measured using Raman spectroscopy. A 785 nm CW laser simultaneously excited Raman scattering and optically heated the sample. The temperature-dependent Stokes peak shift was used to evaluate the actual temperature after calibration [18, 24] (see Figure S6(a) and (b)). Calibration was performed by fitting the experimental temperature-dependent Stokes peak with a generalized four-phonon process reported previously [29] (see Figure S6(c)). The temperature linearly increased with respect to the laser power and the pore size. In addition to experiments, numerical heat transfer simulations and analytical calculations were performed for validation. The former and the latter were based on the finite element method and the heat conduction in an anisotropic medium (Supporting Information, Note 4), respectively. From Figures 4(a), S7 and S8, good agreements were observed between the experimental, numerical, and analytical results that indicate effective thermal conductivity can be used to estimate the temperature increase for the TiN-AAO sample under laser irradiation. Therefore, anisotropic effective thermal conductivity was responsible for the enhanced surface temperature. Furthermore, from the experimental, numerical, and analytical calculations, the slope from the temperature-dependent laser power can be treated as the heating efficiency after dividing by absorptance and extracted for each sample. From Figures 4(b), S9 and S10, it is evident that the higher heating efficiency is dominated by a larger porous structure and not the thickness, as long as the thickness is higher than 40 µm.

Based on these results, it can be concluded that a material with a high heating efficiency can be engineered by combining AAO with plasmonic TiN, where the pore size and thickness of AAO can be systematically controlled by adjusting the anodization and pore-widening times. Our systematic study indicated that a higher heating efficiency is expected for a larger pore size, as long as each pore is not connected. The current results and the analysis methods can be applied to improve the photothermal efficiencies of porous structures for various applications, such as solar water desalination using AAO-based materials and energy harvesting with thermophotovoltaics [30].

## 3 Conclusions

In summary, quantitative heat transfer analyses were conducted for the TiN-AAO samples to enhance the photothermal heating efficiency systematically. In particular, increasing the pore size of AAO using electrochemical anodization can increase its optical absorptance while decreasing its effective thermal conductivity. Upon optimizing the AAO geometry, TiN-AAO with a high heating efficiency can be heated around 400 K with a laser power on the order of few milli-watt. Furthermore, the experimental, numerical simulation, and analytical results agree well with one another, indicating that the effective anisotropic thermal conductivity is responsible for the temperature increase caused by photothermal heating. This study is a potential guide for improving the photothermal heating efficiency of porous materials, which would benefit various applications, such as solar water desalination and thermophotovoltaics.

## 4 Experimental section

### 4.1 TiN-AAO fabrication

AAO was fabricated using aluminum plates of 1 mm thickness (99.99%, KS TRADING) and an electrochemical method. Prior to anodization, Al plates were electropolished to remove the native oxide layer on the surface in a solution containing 2 mL of perchloric acid (60%, Kanto Chemical) and 18 mL of ethanol at room temperature (24 ± 2 °C) and a constant current of 100 mA until the voltage reached 32 V controlled using a source meter (2450, Keithley). Subsequently, an anodization process with 0.3 M phosphoric acid in deionized water at 4 °C was controlled using a cooler (NCB-1210A, Tokyo Rikakikai) for a few hours (1–8 h). Then, pore-widening was performed by dipping the AAO templates into 6 wt % phosphoric acid in deionized water at 30 °C for 1 or 2 h. Finally, 80-nm thick TiN was sputtered on top of the AAO templates by the DC sputtering (CFS-4EP-LL(4G), Shibaura Mechatronics Corp.).

### 4.2 Characterization

The morphologies and reflectance of the samples were characterized using the scanning electron microscope (SEM, S4800, HITACHI) and a UV–Vis spectrometer (BIM-6002, BroLight), respectively. For reflectance measurement, an integrating sphere (ISP-50-8-R-GT, Ocean Optics) was installed to collect the specular and diffusive reflectance owing to the surface roughness of the AAO. A white reflectance standard was used as a reference. In addition, a planar 80-nm thick TiN film was measured with a UV-Vis-NIR spectrometer (SolidSpec-3700, SHIMADZU).

Sample temperatures were characterized using a Raman spectrometer (MicroRAM-300/BZI-1L, Lambda Vision), 785 nm CW laser (laser diode (LD785-SEV300 Thorlabs) powered by a controller (ITC4 002 QCL, Thorlabs) for excitation, and 50 × objective lens (TU Plan Fluor NA0.80, Nikon). The laser spot size was approximately 11 μm in diameter. Initially, a TiN film sputtered on a silicon substrate was placed on a heater (CW-300, Japan High Tech), and Stokes spectra were recorded by gradually increasing the heater temperature at a constant power of 1 mW. The Stokes peak, which was initially at ∼560 cm^−1^, was plotted against the film surface temperature and used as a calibration curve. A polynomial equation which fitted the calibration curve was used to estimate the surface temperatures of the TiN-AAO samples, which were heated by increasing the 785 nm laser power.

### 4.3 Numerical simulations

A commercial software based on the finite element method (COMSOL Multiphysics with wave optics and a heat transfer module) was used to calculate the roughness-dependent absorptance (Supporting Information, Note 1), to derive the effective thermal conductivity of AAO, and to verify the experimental results of the laser-heated samples using heat transfer analysis. Effective thermal conductivities were simulated by defining a unit cell with a hexagonal lattice and applying temperature differences in the x, y, and z directions. The thermal conductivities of air, alumina, and Al were obtained from [31–33], respectively. To simulate the laser-heated surface temperature, a gaussian profile incoming heat flux with a size identical to that of the laser beam was used. AAO structures were homogenized into slabs with simulated effective thermal conductivities and identical sample thicknesses.

### 4.4 Analytical calculations

The heat transfer equation was formulated for a condition wherein one half of the semi-infinite space was an anisotropic material (AAO) and the other half was isotropic (air). The laser spot was expressed as a point source located at the interface. Details can be found in Supplementary Information, Note 4.

## Supplementary Material

Supplementary Material Details
