# Supplementary material for: Photothermal heating and heat transfer analysis of anodic aluminum oxide with high optical absorptance
Source: Nanophotonics. 2022 Jun 14;11(14):3375–81. doi: 10.1515/nanoph-2022-0244 (PMC11501838; doi:10.1515/nanoph-2022-0244)
Supplement: Supplementary file 1 — Supplementary Material Details [file j_nanoph-2022-0244_suppl.pdf]

Supporting Information

Photothermal Heating and Heat Transfer Analysis of Anodic Aluminum Oxide with High Optical Absorptance

Nicholaus Kevin Tanjaya[1,2], Manpreet Kaur[1], Tadaaki Nagao[1,3], Satoshi Ishii[1,2,4,*]

[1]International Center for Materials Nanoarchitectonics (MANA), National Institute for Materials Science (NIMS), Tsukuba, Ibaraki, 305-0044, Japan

[2]Faculty of Pure and Applied Physics, University of Tsukuba, Tsukuba, Ibaraki, 305-8577, Japan

[3]Department of Condensed Matter Physics, Graduate School of Science, Hokkaido University, Sapporo, Hokkaido, 060-0810, Japan.

[4]PRESTO, Japan Science and Technology Agency, Kawaguchi, Saitama, 332-0012, Japan

E-mail: sishii@nims.go.jp

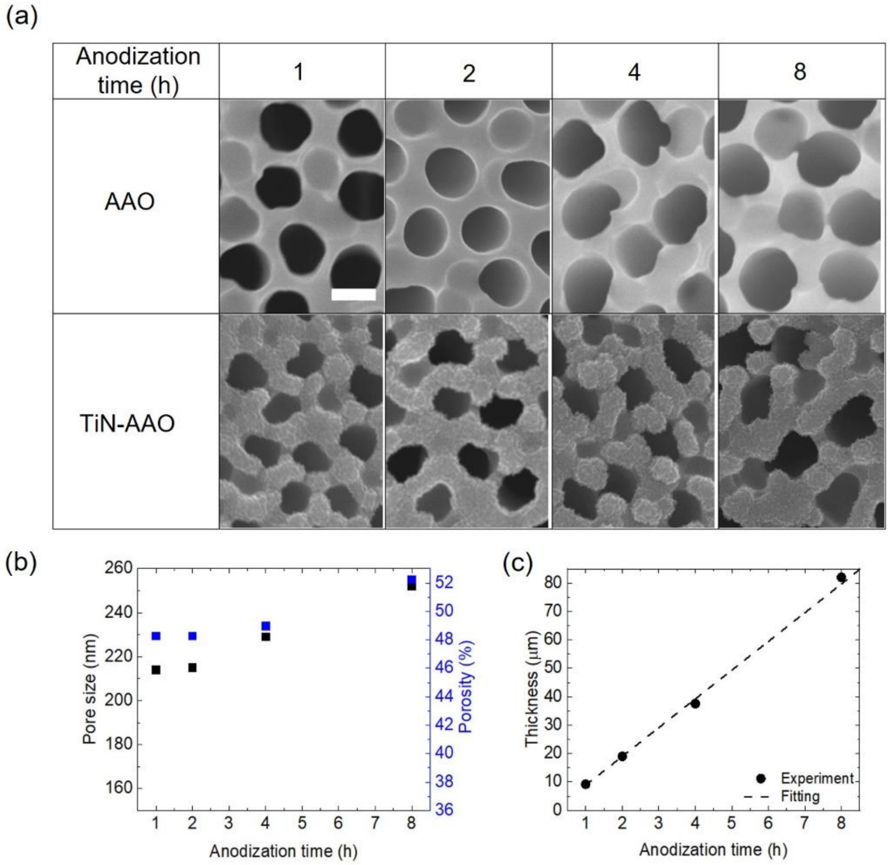

Figure S1. AAO and TiN-AAO morphology for pore widening 2 h samples. (a) Scanning electron microscope images of the samples anodized for a different time. Scale bar 200 nm. (b) Anodization time-dependent pore size (square) and porosity (circle), with error from standard deviation, is 30 nm. (c) Anodization-time-dependent thickness.

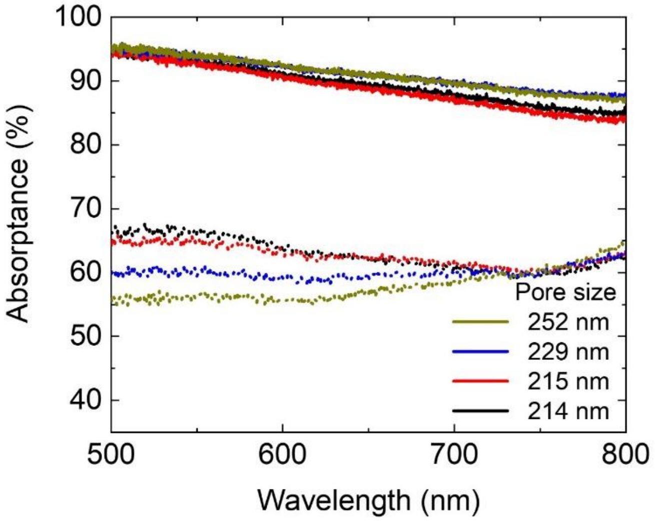

Figure S2. UV-Vis spectroscopy for samples pore widened for 2 h. Bold and dotted lines corresponds to TiN-AAO samples and AAO samples, respectively.

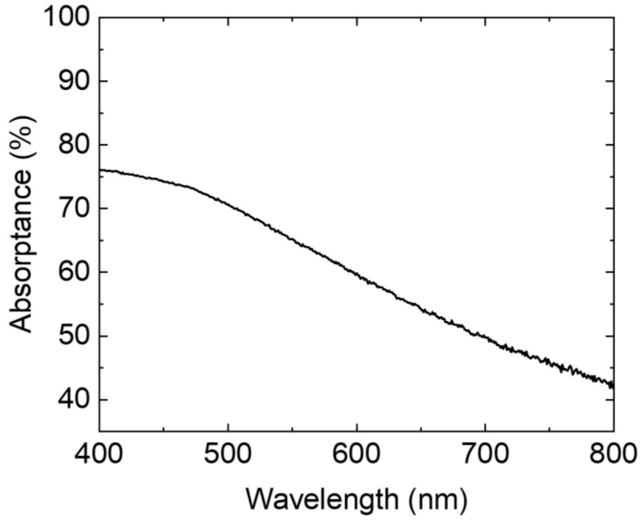

Figure S3. Absorptance spectrum of 80-nm thick planar TiN film on a glass substrate.

Note 1. Numerical analysis of the roughness effect in reflectance between aluminum and AAO

A commercial finite element method software (COMSOL Multiphysics) was used in the numerical simulation to calculate absorptance. The unit cell has a hexagonal lattice and consists of air, AAO, and aluminum layers where the thickness of AAO was one micrometer. The roughness was introduced between the AAO and aluminum. Three possible configurations were simulated; alumina on planar aluminum substrate, perfectly periodic AAO on planar aluminum, and randomly arranged AAO with surface roughness between AAO and Al interface. The comparison is presented in Figure S4. The results show an increase in enhancement due to surface roughness between AAO and Al interface.

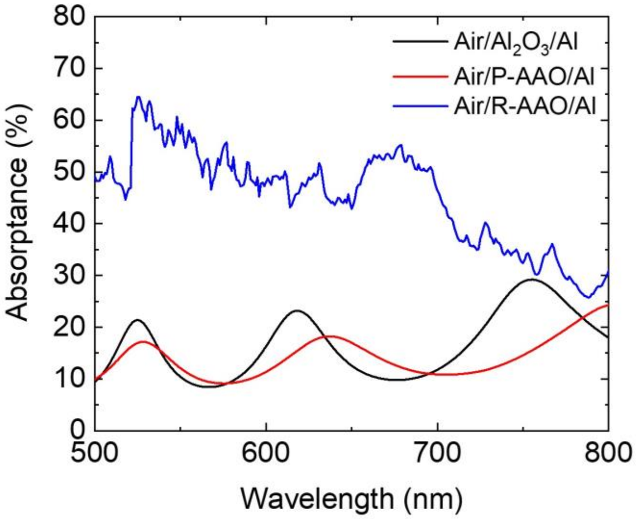

Figure S4. Numerical calculation of absorptance spectrum from air/alumina/aluminum configuration. Three different designs were simulated; Air/Al_2O_3/Al, Air/P-AAO/Al, and Air/R-AAO/Al correspond to alumina on planar Al substrate, perfectly periodic AAO on planar Al, and randomly arranged AAO with surface roughness between AAO and Al interface, respectively.

Note 2. Comparison of effective thermal conductivities calculated by Maxwell's effective media theory and finite element method

From Maxwell's effective media theory, the effective thermal conductivity in all 3D can be calculated with known bulk thermal conductivity (k_s) and the porosity (ϕ). Effective thermal conductivity perpendicular to the porous axis ($k_\mathrm{x}, k_\mathrm{y}$) and parallel to the porous axis (k_z) are calculated using Eqs. (1) and (2), respectively. The effectively thermal conductivities calculated from the Maxwell's effective media theory[1] and finite element method are compared in Figure S5. Within the porosity range plotted in the figure, the values from the two different methods match well.

$$k_\mathrm{x} = k_\mathrm{y} = k_\mathrm{s}\frac{(1-\phi)}{(1+\phi)} \quad (1)$$

$$k_\mathrm{z} = k_\mathrm{s}(1-\phi) \quad (2)$$

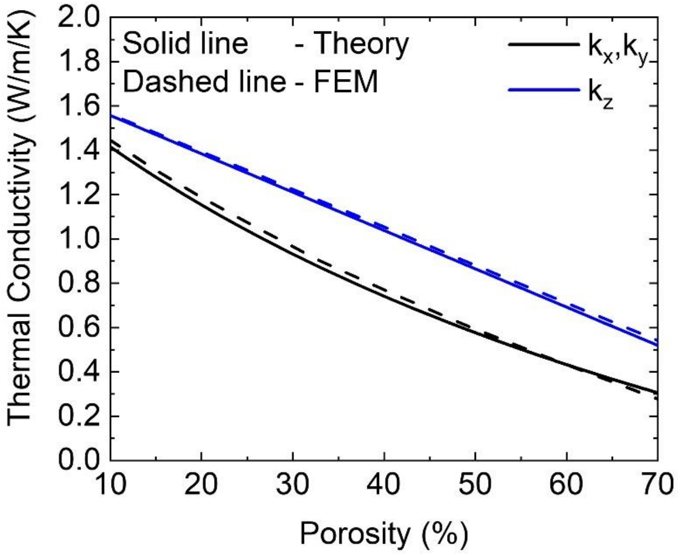

Figure S5. Comparison of effective thermal conductivities calculated from Maxwell's effective media theory and finite element method (FEM). The data from the finite element method is identical to the data plotted in Figure 3(b) in the main text.

Note 3. Temperature-dependent Stokes peak shift

As explained briefly in the experimental section (characterization) of main text, the temperature was converted from the temperature-dependent Stokes peak shift by recording the Raman spectra at different sample temperatures controlled with a heater at a constant laser power (~1 mW). The temperature dependent Stokes peak shift are shown in the Figure S6(a). Then, the Stokes peak was plotted against the film surface temperature, and fitted with a generalized four-phonon process equation[2] (Eq. (3)) as shown in Figure S6(b) where Ω, ω_0, $\hbar$, k_B, and T are the temperature-dependent Raman Stokes peak, Stokes peak, Planck constant, Boltzmann constant, and temperature, respectively. Finally, the fitted calibration curve was inversed and further fitted with

fourth-order polynomial (Eq. (4)) to get the relation between the temperature and Stokes peak shift as shown in Figure S6(c).

$$\Omega(T) = \omega_0 + A\left[1 + \frac{2}{e^x - 1}\right] + B\left[1 + \frac{3}{e^y - 1} + \frac{3}{(e^y - 1)^2}\right], x = \frac{\hbar\omega_0}{2k_BT}; y = \frac{\hbar\omega_0}{3k_BT}, \quad (3)$$

$$T(\Delta\Omega) = A + B(\Delta\Omega) + C(\Delta\Omega)^2 + D(\Delta\Omega)^3 + E(\Delta\Omega)^4. \quad (4)$$

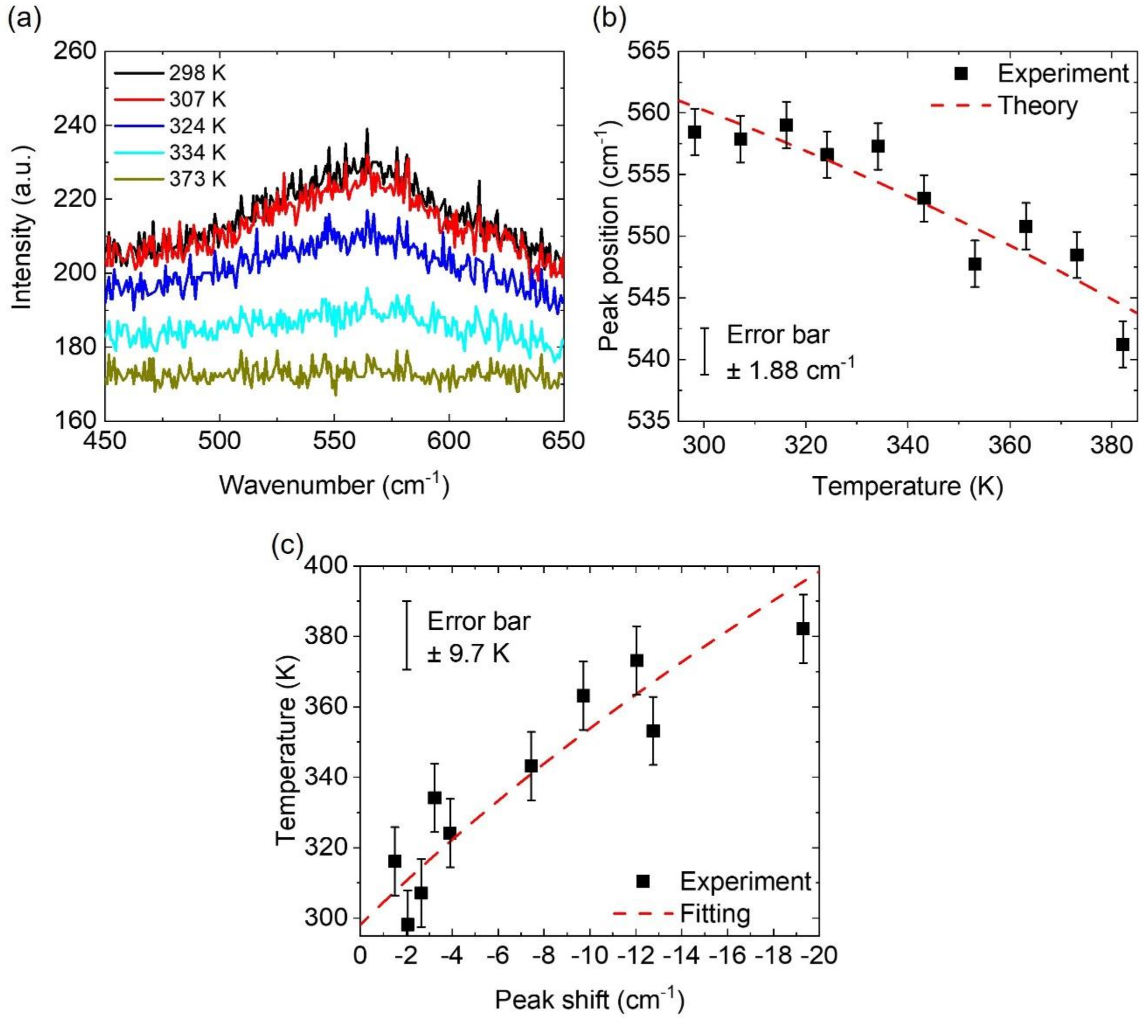

Figure S6. Temperature extraction process from Temperature dependent Stokes peak shift. (a) TiN Raman spectrum at different temperature. (b) Temperature dependent Stokes peak extracted from Raman spectrum in (a) fitted with four-phonon process. (c) Peak-shift-dependent temperature fitted with fourth-order polynomial function. The error bars in (b) and (c) are defined from the mean difference of the experiment data with the four-phonon process fit.

Note 4. Analytical calculation of the laser-power-dependent temperature

The problem from the TiN-AAO was approached with analytical modeling following the derivation in the reference[3]. Considering a point source at the origin of a cartesian coordinate system where the surrounding area has anisotropic thermal conductivity.

Transformed heat transfer equation for steady-state problem in an anisotropic medium can be expressed as follows.

$$\frac{\partial^2 T}{\partial X^2} + \frac{\partial^2 T}{\partial Y^2} + \frac{\partial^2 T}{\partial Z^2} = 0\,; \begin{cases} 0 < X < \infty \\ 0 < Y < \infty, \\ 0 < Z < \infty \end{cases} \tag{5}$$

where X, Y, Z is the transformed coordinate from cartesian coordinate and k is a reference conductivity from thermal conductivity in each x, y, and z-axis (k_x, k_y k_z) as follows,

$$X = \left(\frac{k}{k_x}\right)^{\frac{1}{2}} x; Y = \left(\frac{k}{k_y}\right)^{\frac{1}{2}} y; Z = \left(\frac{k}{k_z}\right)^{\frac{1}{2}} z \tag{6}$$

$$k = \left(k_x k_y k_z\right)^{\frac{1}{3}} . \tag{7}$$

The boundary condition for the system is obtained by making an imaginary sphere with a radius R located at the origin in the transformed coordinate, which calculates the rate of energy released from the point source and conducted to the medium.

$$(4\pi R^2)\left(-k\frac{\partial T}{\partial R}\right) = Q; R \to 0\ , \tag{8}$$

where the radius R is calculated as follow,

$$R = (X^2 + Y^2 + Z^2)^{\frac{1}{2}} . \tag{9}$$

The boundary at infinity is that the temperature reaches a finite temperature (T_∞).

$$T = T_\infty; R \to \infty\ . \tag{10}$$

The solution of the equation (5) satisfying the boundary condition is,

$$T(R) = \frac{C}{R} + T_\infty\ . \tag{11}$$

where C is a constant determined from the boundary condition,

$$(4\pi R^2)\left(k\frac{C}{R^2}\right) = Q \to C = \frac{1}{k}\frac{Q}{4\pi}\ . \tag{12}$$

Substituting the constant C to the equation (11), then the solution for the problem is,

$$T(R) = \frac{Q}{4\pi}\frac{1}{kR} + T_\infty. \tag{13}$$

However, while the above derivation assumes a point source located in a homogeneous medium, in practice, the point source is located at the interface of the materials. Therefore, the thermal conductivity (k) is substituted with the average thermal conductivity of the superstrate and substrate ($k_{\text{avg}} = k_{\text{superstrate}} + k_{\text{substrate}}/2$). In addition, the heat source has a gaussian profile. Thus, the solution in equation (13) transforms to,

$$T(R) = \frac{Q}{4\pi}\frac{1}{k_{\text{avg}}R}e^{-\frac{2R^2}{w^2}} + T_{\infty} \tag{14}$$

where w is the laser spot size. Afterward, the temperature distribution in equation (14) is averaged from the origin to R.

$$T_{\text{avg}} = \frac{1}{\pi R^2}\iint T(R)RdRd\theta \tag{15}$$

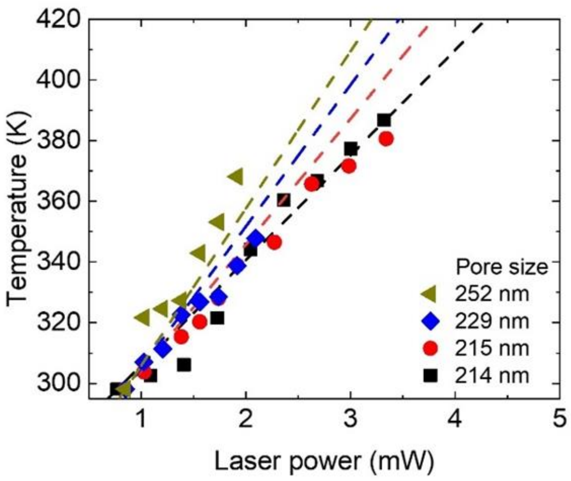

Figure S7. Laser-power-dependent temperature comparison between experiments (symbol) and numerical simulations (dashed line) for 2 h pore-widened samples.

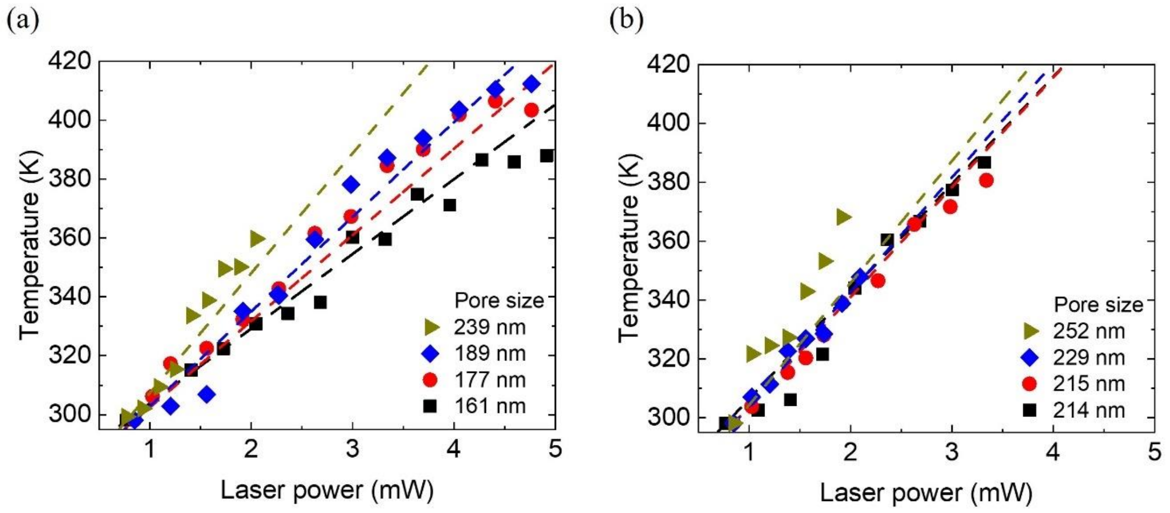

Figure S8. Laser-power-dependent temperature comparison between experiments (symbol) and analytical calculations (dashed line) for (a) 1 h and (b) 2 h pore-widened samples. The experimental data presented in sub-panels (a) and (b) are identical to the ones presented in Figure 4(a) and S7, respectively.

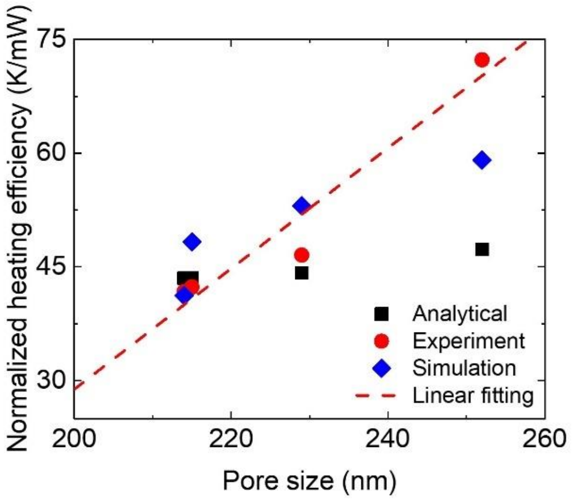

Figure S9. Photothermal heating efficiency of the experiment, simulation, and analytical data for TiN-AAO samples that were pore widen for 2 h. The linear fitting was from the experiment results.

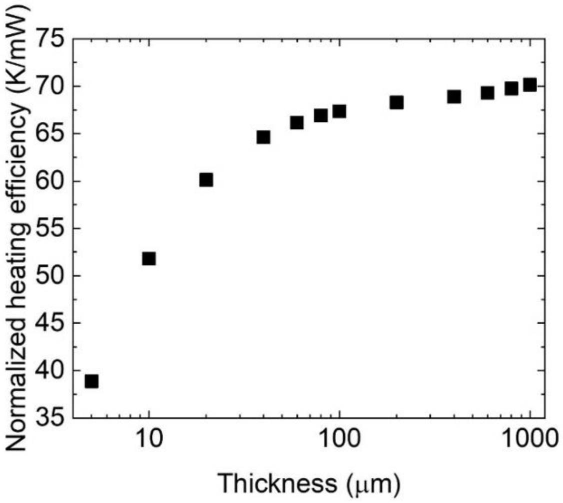

Figure S10. Simulated thickness-dependent heating efficiency normalized with absorptance and fixed porosity of 50 %.

References

1. Maxwell, J.C., *A treatise on electricity and magnetism*. 1873, Oxford: Clarendon Press.
2. Balkanski, M., R.F. Wallis, and E. Haro, *Anharmonic effects in light scattering due to optical phonons in silicon.* Phys. Rev. B, 1983. **28**(4): p. 1928-1934.
3. David W. Hahn, M.N.Ö., *Heat Conduction in Anisotropic Solids*, in *Heat Conduction*. 2012. p. 614-650.
